# *Mycobacterium marinum* MMAR_2380, a predicted transmembrane acyltransferase, is essential for the presence of the mannose cap on lipoarabinomannan

**DOI:** 10.1099/mic.0.037507-0

**Published:** 2010-11

**Authors:** Nicole N. Driessen, Esther J. M. Stoop, Roy Ummels, Sudagur S. Gurcha, Arun K. Mishra, Gérald Larrouy-Maumus, Jérôme Nigou, Martine Gilleron, Germain Puzo, Janneke J. Maaskant, Marion Sparrius, Gurdyal S. Besra, Wilbert Bitter, Christina M. J. E. Vandenbroucke-Grauls, Ben J. Appelmelk

**Affiliations:** 1 Department of Medical Microbiology and Infection Control, VU University Medical Center, 1081 BT Amsterdam, The Netherlands; 2 School of Biosciences, University of Birmingham, Edgbaston B15 2TT, UK; 3 CNRS, IPBS (Institut de Pharmacologie et de Biologie Structurale), 205 Route de Narbonne, F-31077 Toulouse, France; 4 Université de Toulouse, UPS, IPBS, F-31077 Toulouse, France

## Abstract

Lipoarabinomannan (LAM) is a major glycolipid in the mycobacterial cell envelope. LAM consists of a mannosylphosphatidylinositol (MPI) anchor, a mannan core and a branched arabinan domain. The termini of the arabinan branches can become substituted with one to three *α*(1→2)-linked mannosyl residues, the mannose cap, producing ManLAM. ManLAM has been associated with a range of different immunomodulatory properties of *Mycobacterium tuberculosis* during infection of the host. In some of these effects, the presence of the mannose cap on ManLAM appears to be crucial for its activity. So far, in the biosynthesis of the mannose cap on ManLAM, two enzymes have been reported to be involved: a mannosyltransferase that adds the first mannosyl residue of the mannose caps to the arabinan domain of LAM, and another mannosyltransferase that elongates the mannose cap up to three mannosyl residues. Here, we report that a third gene is involved, *MMAR_2380*, which is the *Mycobacterium marinum* orthologue of *Rv1565c*. *MMAR_2380* encodes a predicted transmembrane acyltransferase. In *M. marinum* Δ*MMAR_2380*, the LAM arabinan domain is still intact, but the mutant LAM lacks the mannose cap. Additional effects of mutation of *MMAR_2380* on LAM were observed: a higher degree of branching of both the arabinan domain and the mannan core, and a decreased incorporation of [1,2-^14^C]acetate into the acyl chains in mutant LAM as compared with the wild-type form. This latter effect was also observed for related lipoglycans, i.e. lipomannan (LM) and phosphatidylinositol mannosides (PIMs). Furthermore, the mutant strain showed increased aggregation in liquid cultures as compared with the wild-type strain. All phenotypic traits of *M. marinum* Δ*MMAR_2380*, the deficiency in the mannose cap on LAM and changes at the cell surface, could be reversed by complementing the mutant strain with *MMAR_2380*. Strikingly, membrane preparations of the mutant strain still showed enzymic activity for the arabinan mannose-capping mannosyltransferase similar to that of the wild-type strain. Although the exact function of MMAR_2380 remains unknown, we show that the protein is essential for the presence of a mannose cap on LAM.

## INTRODUCTION

The complex mycobacterial cell envelope contains an exceptionally high amount of lipids. One of the major glycolipids present is lipoarabinomannan (LAM) (Briken *et al.*, 2004; Chatterjee & Khoo, 1998; Nigou *et al.*, 2002, 2003). With its mannosylphosphatidylinositol (MPI) anchor, LAM is probably inserted in both the cytoplasmic membrane and the outer membrane (Hoffmann *et al.*, 2008; Pitarque *et al.*, 2008). The glycan portion of LAM comprises a mannan core, which consists of *α*(1→6)-linked mannosyl residues substituted at some positions with single *α*(1→2)-linked mannoses, and an arabinan domain consisting of *α*(1→5)-linked arabinose (see Supplementary Fig. S1). This latter domain is branched with side chains that always end in *β*(1→2)-linked arabinosyl units (AraLAM). In mannose-capped LAM (ManLAM), a part of these terminal arabinosyl residues is substituted with an *α*(1→5)-linked mannosyl residue which can be further elongated by the addition of one or two *α*(1→2)-linked mannosyl residues, forming the mannose cap (Supplementary Fig. S1). Whether mannose caps are present on LAM depends on the *Mycobacterium* species (Pitarque *et al.*, 2005).

Several immunomodulating properties of LAM have been reported which could codetermine the host–pathogen interaction during infection with pathogenic mycobacteria, such as *Mycobacterium tuberculosis*. Some of these have been linked specifically to the presence of the mannose cap on LAM, e.g. the binding of ManLAM to pattern recognition receptors such as the dendritic cell-specific intercellular adhesion molecule-3-grabbing nonintegrin (DC-SIGN) (Geijtenbeek *et al.*, 2003; Maeda *et al.*, 2003) and the macrophage mannose receptor (MMR) (Schlesinger *et al.*, 1994), and its role in phagosome maturation arrest (Kang *et al.*, 2005; Welin *et al.*, 2008).

So far, two mannosyltransferases have been identified as being involved in the biosynthesis of the mannose cap on ManLAM. The first, encoded by *Rv1635c*, adds the first mannosyl residue of the mannose cap to the non-reducing arabinan termini (Dinadayala *et al.*, 2006). *Rv2181* encodes the second, cap-elongating mannosyltransferase, which besides lengthening of the mannose cap, substitutes the mannan core of LAM with the single *α*(1→2)-linked mannose moieties (Kaur *et al.*, 2006, 2008). Both enzymes use polyprenylphosphomannose (PPM) as a sugar donor (Appelmelk *et al.*, 2008; Kaur *et al.*, 2006). A potential glycosyltransferase function of these enzymes has been predicted using bioinformatics tools. Although very successful, this approach is biased towards finding glycosyltransferases directly involved in the biosynthesis of glycolipids or glycoproteins, and proteins that exert more unexpected functions may be missed.

In an alternative approach to identify novel genes with a role in the biosynthesis of ManLAM, we have used a colony blot screen of a transposon-insertion mutant library in *Mycobacterium marinum* in which single-transposon mutants were probed with mannose cap-recognizing mAb 55.92.1A1 (Appelmelk *et al.*, 2008). As recently described, this screen yielded the mutant in *MMAR_2439*, the *M. marinum* orthologue of *Rv1635c*. Subsequent mannosyltransferase assays and chemical analysis confirmed that the function of *MMAR_2439* is the addition of the first mannosyl residue of the mannose cap to LAM, identical to *Rv1635c* (Appelmelk *et al.*, 2008).

We have extended our search using the screening approach with the mannose cap-recognizing mAb. In this paper, we reveal a third gene involved in the biosynthesis of the mannose cap on LAM. In *M. marinum* Δ*MMAR_2380*, the LAM molecule is still intact, apart from lacking its mannose cap. Additional effects of the mutation of *MMAR_2380* were seen in a higher degree of branching of both the arabinan domain and the mannan core in mutant LAM, decreased [1,2-^14^C]acetate incorporation in the acyl chains of mutant LAM and related lipoglycans, and an altered cell surface of the mutant strain as compared with wild-type *M. marinum*. *MMAR_2380* encodes a predicted transmembrane acyltransferase, and here we explored potential roles for MMAR_2380 in the biosynthesis of LAM in *M. marinum*.

## METHODS

### Bacterial strains and growth conditions.

*M. marinum* strain E11 (Puttinaowarat *et al.*, 1999) and *Mycobacterium smegmatis* mc^2^155 were grown in Middlebrook 7H9 broth (Difco) with 10 % Middlebrook albumin dextrose catalase (ADC) enrichment (BBL) and 0.05 % (v/v) Tween 80, or on Middlebrook 7H10 agar (Difco) with 10 % Middlebrook oleic acid albumin dextrose catalase (OADC) enrichment (BBL) at 30 and 37 °C, respectively. *Escherichia coli* DH5*α* was grown on LB at 37 °C. Mycobacterial growth was measured by determining OD_600_ with a UV/visible spectrophotometer (Jenway). The concentrations of antibiotics used were 25 μg kanamycin ml^−1^ and 50 μg hygromycin ml^−1^ for mycobacteria, and 100 μg hygromycin ml^−1^ for *E. coli*.

### Immunoblotting assay.

Bacteria were grown until the late-exponential phase and disrupted with a Beadbeater (BioSpec). Protein concentration was measured with a bicinchoninic acid (BCA) Protein Assay (Pierce). Supernatants were run on a 10 % SDS-PAGE gel and immunostained after electroblotting on a PVDF membrane (Millipore). Murine mAbs of the IgM class were used. mAb F30-5 recognizes the branches of the arabinan domain of LAM (Appelmelk *et al.*, 2008; Kolk *et al.*, 1984). mAb 55.92.1A1 binds the mannose cap on ManLAM (Appelmelk *et al.*, 2008). First the PVDF membranes were incubated in 0.5 % (w/v) blocking reagent (Boehringer) for 1 h at 37 °C, followed by another incubation of 2 h with the mAb diluted in 1 : 1 blocking buffer and PBS+0.05 % (v/v) Tween 80 (PBST). After thorough washing in PBST, the membranes were incubated with horseradish peroxidase (HRP)-labelled goat anti-mouse IgM (American Qualex) in PBST with 0.5 % (v/v) normal goat serum. After washing, the membranes were stained with 4-chloro-1-naphthol (4-CN; Bio-Rad) and 3,3′-diaminobenzidine.4HCl (DAB; Sigma).

### Identification of a novel *M. marinum* mutant deficient in the biosynthesis of the mannose cap on LAM.

A transposon insertion mutant library was created in *M. marinum* E11 with the Himar1-based mariner transposon, which has little insertion site specificity (dinucleotide TA) (Rubin *et al.*, 1999). The transposon was introduced via phagemid ϕMycoMarT7, as described by Sassetti *et al.* (2001). Mutants selected on kanamycin were screened in a colony blot assay for the absence of the mannose cap on LAM using mAb 55.92.1A1, as described previously (Appelmelk *et al.*, 2008). In brief, single colonies were transferred to PVDF membranes with sterile toothpicks, after which the membranes were baked for 1 h at 70 °C and subsequently treated as in the immunoblotting assay described above. Mutants that were repeatedly negative with 55.92.1A1 in colony blots were examined further by SDS-PAGE and immunostaining. The location of the transposon insertion was determined by ligation-mediated PCR, as described previously (Abdallah *et al.*, 2006). The exact insertion position of the transposon in gene *MMAR_2380* was confirmed by standard PCR with the pMyco1-primer and a second primer, cap-RV, which binds about 210 bp downstream from *MMAR_2380*. The PCR product was sequenced on an ABI Prism 300 DNA sequencer (Applied Biosystems). To determine the direction of the kanamycin-resistance cassette in the transposon in *MMAR_2380*, two primers were used, MMAR_2380-FW and MMAR_2380-RV, with a binding site upstream or downstream of the transposon in gene *MMAR_2380*, respectively, and two primers binding to sequences within the transposon (the kanamycin cassette), Kana-FW and Kana-RV. See Supplementary Table S1 for primer sequences.

### Complementation of the *M. marinum* Δ*MMAR_2380* mutant strain.

*MMAR_2380* was amplified from genomic *M. marinum* E11 wild-type DNA with primers cap-FW and cap-RV (Supplementary Table S1) using the Expand High Fidelity PCR kit (Roche). The obtained PCR product (2973 bp) was cloned into pCRII-TOPO. This plasmid was digested with *Hin*dIII and *Eco*RV and the fragment was ligated into *Hin*dIII/*Eco*RV-digested pSMT3-eGFP (Abdallah *et al.*, 2006). In the resulting plasmid (pSMT3-*eGFP*-*MMAR_2380*), the gene of interest was located behind the GFP gene under the control of heat-shock promoter 60 (HSP60). The complementation plasmid was isolated from *E. coli* DH5*α* cells using the QIAprep Miniprep kit (Qiagen) and electroporated into *M. marinum* Δ*MMAR_2380*. Transformants (*M. marinum* Δ*MMAR_*2380 comp.) were selected on hygromycin.

### Chemical analysis of the mannose cap on LAM.

The presence of the mannose cap in *M. marinum* wild-type and *M. marinum* Δ*MMAR_2380* strains was analysed by capillary electrophoresis (CE), as described previously (Appelmelk *et al.*, 2008; Nigou *et al.*, 2000). In brief, purified LAM was partially degraded by controlled acid hydrolysis (0.1 M HCl for 20 min at 110 °C), and the oligosaccharides liberated were tagged with the fluorescent label 8-aminopyrene-1,3,6-trisulfonate (APTS). During CE, the labelled oligosaccharides were separated and peaks detected by laser-induced fluorescence, and elution times were compared with the appropriate standards.

### Mannosyltransferase assay.

Membranes of *M. marinum* E11 wild-type, and the Δ*MMAR_2380* and Δ*MMAR_2380* complemented strains were prepared as follows: mycobacterial cells (10 g wet weight) were washed and resuspended in 30 ml buffer A, containing 50 mM MOPS (adjusted to pH 8.0 with KOH), 5 mM *β*-mercaptoethanol and 10 mM MgCl_2_ at 4 °C and subjected to probe sonication (Soniprep 150, MSE Sanyo; 1 cm probe) for a total time of 10 min in 60 s pulses with 90 s cooling intervals between pulses. The sonicate was centrifuged at 27 000 ***g*** for 20 min at 4 °C. Membrane fractions were obtained by centrifugation of the clarified lysate at 100 000 ***g*** for 1 h at 4 °C. The supernatant was carefully removed and the membranes gently resuspended in buffer A at a protein concentration of 20 mg ml^−1^. Protein concentrations were determined using the BCA Protein Assay Reagent kit (Pierce) (Lee *et al.*, 1998). Assay conditions consisted of buffer A, 1 mM ATP, membranes (500 μg), 0.1 mM decaprenyl phosphate (stored in 1 % CHAPS as a 1 mM stock solution), acceptor Ara_6_ (1 mM) and GDP-[^14^C]mannose [0.25 μCi (9.25 kBq), 11 GBq mmol^−1^, Amersham] to a final reaction volume of 120 μl. Ara_6_ is synthetic aminooctyl-linked hexaarabinofuranoside, a motif representing the terminal, non-reducing part of LAM (Gadikota *et al.*, 2003). The reaction mixtures were then incubated at 37 °C for 1 h. The enzymically synthesized products were extracted using ‘E-soak’ (water : ethanol : diethyl-ether : pyridine : ammonium hydroxide; 15 : 15 : 5 : 1 : 0.017 by vol.), which was added to the incubation tubes. These were mixed for 30 min and then centrifuged at 18 000 ***g***. The supernatant was recovered and dried under reduced pressure with a Genevac EZ-2 evaporator, resuspended in water (1 ml) and loaded onto a pre-equilibrated Alltech C18 Sep-Pak cartridge (600 mg). The cartridge was washed with 5 ml water, and the enzymically synthesized products were eluted with methanol (5 ml) and subsequently dried under vacuum as described above. The dried eluate was resuspended in water (1 ml) and loaded onto a pre-equilibrated Supelco strong anion exchange (SAX) cartridge, which was washed with 5 ml water. The water eluate was collected, dried under vacuum and resuspended in 200 μl water, and contained the ^14^C-mannosylated acceptor Ara_6_ product. The total c.p.m. of recovered radiolabelled material with this two-step separation protocol was measured by scintillation counting of 10 % of the labelled material in 10 ml EcoScintA (National Diagnostics). The incorporation of [^14^C]mannose was determined by subtracting counts present in control assays (reaction components incubated in the absence of acceptor Ara_6_) (Appelmelk *et al.*, 2008).

### NMR analysis of the mannose cap, arabinan domain and mannan core of LAM.

NMR spectra of LAM from *M. marinum* wild-type and *M. marinum* Δ*MMAR_2380* were recorded on a Bruker DMX-500 NMR spectrometer equipped with a double resonance (1H/X)-BBi *z*-gradient probe head. All samples were exchanged in D_2_O (99.97 % D, Euriso-top), with intermediate lyophilization, and then dissolved in 0.5 ml D_2_O and analysed at 313 K. The ^1^H and ^13^C NMR chemical shifts were referenced relative to internal acetone at 2.225 and 34.00 p.p.m. respectively. All the details concerning NMR sequences used and experimental procedures have been described in previous studies (Gilleron *et al.*, 2000).

### Glycosidic linkage analysis of LAM.

Glycosyl linkage composition was performed according to the modified procedure of Ciucanu & Kerek (1984). The per-*O*-methylated LAM was hydrolysed using 500 μl 2 M trifluoroacetic acid (TFA) at 110 °C for 2 h, reduced using 350 μl 10 mg ml^−1^ sodium borodeuteride [NaBD_4_ (1 M NH_4_OH : C_2_H_5_OH, 1 : 1, v/v)] and per-*O*-acetylated using 300 μl acetic anhydride for 1 h at 110 °C. The resulting alditol acetates were solubilized in cyclohexane before analysis by GC and GC/MS.

### [1,2-^14^C]Acetate incorporation in total lipids.

*M. marinum* strains were grown as described above and metabolically labelled using 5 μCi ml^−1^ [1,2-^14^C]acetate [50–62 mCi mmol^−1^(1850–2294 MBq mmol^−1^), GE Healthcare, Amersham Bioscience] at OD_600_ 0.4, and cultures were grown for a further 4 h at 37 °C with gentle shaking. Cells were harvested by centrifugation and washed once with PBS, and a small-scale apolar and polar lipid extraction was performed as described by Dobson *et al.* (1985). The lipid extracts were dried and resuspended in CHCl_3_ : CH_3_OH (2 : 1), and equal amounts of crude lipid (25 000 c.p.m.) were applied to the corners of 6.6×6.6 cm pieces of Merck 5554 aluminium-backed TLC plates. The plates were developed using solvent system E for polar lipids: chloroform : methanol : water (60 : 30 : 6, by vol.) in the first direction, and chloroform : acetic acid : methanol : water (40 : 25 : 3 : 6, by vol.) in the second direction (Besra, 1998; Dobson *et al.*, 1985). Plates were dried and autoradiograms were produced by overnight exposure of Kodak X-Omat AR film to the TLC plates to reveal [1,2-^14^C]acetate-labelled lipids.

### [1,2-^14^C]Acetate incorporation in lipoglycans.

Lipoglycans were extracted from [1,2-^14^C]acetate-labelled delipidated cells as previously described (Ludwiczak *et al.*, 2001). Briefly, cells were broken by sonication (MSE Soniprep 150, 12 μm amplitude, 60 s on, 90 s off for 10 cycles, on ice) and the cell debris was refluxed five times with 50 % C_2_H_5_OH at 68 °C, for 12 h intervals. The cell debris was removed by centrifugation and the supernatant containing lipoglycans and neutral glycans was dried. The dried extract was then treated with 80 % hot phenol at 85 °C. The aqueous phase was dialysed and dried, followed by extensive treatments with *α*-amylase, DNase, RNase, chymotrypsin and trypsin to degrade any contamination. This fraction was dialysed to remove the low-molecular-mass breakdown products formed after the enzyme treatment, thus yielding the crude lipoglycan fraction. The crude lipoglycan extract was dried and resuspended in buffer A (50 mM ammonium acetate and 15 % propan-1-ol) and subjected to Octyl Sepharose CL-4B chromatography. The column (2.5×50 cm) was washed initially with four column volumes of buffer A to ensure removal of neutral glycans, followed by buffer B (50 mM ammonium acetate and 50 % propan-1-ol). The eluent was collected and concentrated to approximately 1 ml and precipitated using 5 ml C_2_H_5_OH. The sample was dried using a Savant Speedvac concentrator and resuspended in water, and the incorporation of [1,2-^14^C]acetate into lipoglycans was determined. Furthermore, 25 μg lipoglycans was loaded on a 15 % SDS-PAGE gel and developed using autoradiograms, as described previously (Mishra *et al.*, 2008).

## RESULTS AND DISCUSSION

### *M. marinum* MMAR_2380 is essential for the presence of the mannose cap on LAM

Two genes have been reported to be involved in the biosynthesis of the mannose cap on ManLAM, *Rv1635c* and *Rv2181* (Dinadayala *et al.*, 2006; Kaur *et al.*, 2008). An *M. marinum* mutant strain in which the homologue of *Rv1635c* (*MMAR_2439*) is mutated has also been isolated in a colony blot screen with mannose cap-recognizing mAb 55.92.1A1 (anti-ManLAM; *α*-ManLAM) (Appelmelk *et al.*, 2008).

**Fig. 1.**
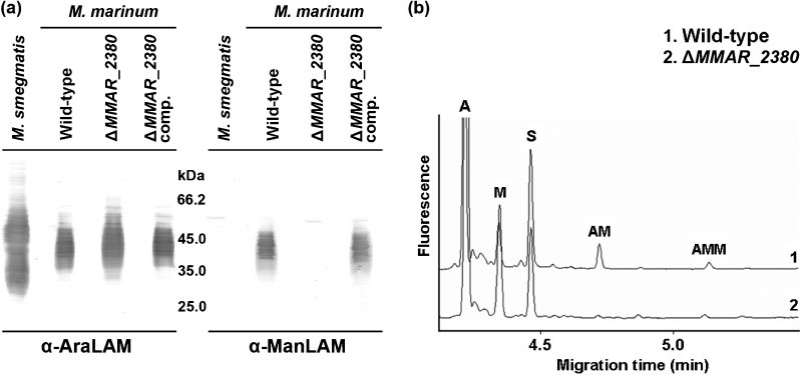
Absence of the mannose cap on mutant LAM. (a) Cell lysates from *M. marinum* wild-type, Δ*MMAR_2380* and complemented Δ*MMAR_2380* (Δ*MMAR_2380* comp.) strains and *M. smegmatis* wild-type (control for ‘capless’ LAM) were immunoblotted with *α*-AraLAM antibody F30-5, which recognizes LAM, and *α*-ManLAM antibody 55.92.1A1, which recognizes the mannose cap. (b) Mannooligosaccharide cap analysis of LAM. Purified and partially degraded LAM was analysed for the presence of the mannose caps by CE. Shown are the profiles of LAM purified from *M. marinum* E11 (trace 1) and *M. marinum* Δ*MMAR_2380* (trace 2). A, Ara-APTS; M, Man-APTS; S, internal standard, mannoheptose-APTS; AM, Manp-*α*(1→5)-Ara-APTS (monomannoside cap); AMM, Manp-*α*(1→2)-Manp-*α*(1→5)-Ara-APTS (dimannoside cap).

By continuing with this screen, we identified a novel gene involved in mannose-capping. LAM from an *M. marinum* mutant strain with a transposon inserted in *MMAR_2380* at 91 bp upstream of its 3′ end (Supplementary Fig. S2) reacted well with an anti-AraLAM (*α*-AraLAM) mAb (mAb F30-5) which recognizes the arabinan branches of LAM, but not with *α*-ManLAM (Fig. 1a). The absence of the mannose cap on LAM in *M. marinum* Δ*MMAR_2380* was further verified by chemical analysis. Both the mono- and di-mannoside caps, which are the dominant cap-lengths in the wild-type strain of *M. marinum* (Pitarque *et al.*, 2005), were not detectable in the mutant strain (Fig. 1b). By complementing *M. marinum* Δ*MMAR_2380* with a wild-type copy of *MMAR_2380* (region indicated in Supplementary Fig. S2), the biosynthesis of the mannose cap on LAM was restored (Fig. 1a). This also excluded the possibility that the transposon affected the *treXYZ*-operon located downstream of *MMAR_2380* rather than *MMAR_2380* (Supplementary Fig. S2).

### Mannosyltransferase capping enzymic activity is still present in the *M. marinum* Δ*MMAR_2380* mutant strain

**Fig. 2.**
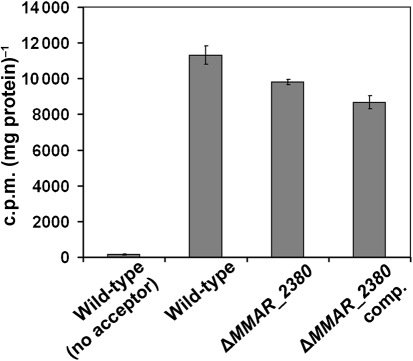
Enzymic analysis. Mannosyltransferase capping assay with synthetic Ara_6_ as acceptor and membranes from *M. marinum* wild-type, Δ*MMAR_2380* and complemented Δ*MMAR_2380* (Δ*MMAR_2380* comp.), and as control, membranes from *M. marinum* wild-type without acceptor. Shown are means of triplicates; error bars, sd.

Since the full-length mannose cap is missing in the mutant strain, we checked the potential capability of the latter to add the first mannosyl unit of the cap. We used a mannosyltransferase assay previously set up to characterize the enzymic activity of the *MMAR_2439* (*Rv1635c*) gene product (Appelmelk *et al.*, 2008). A membrane preparation from *M. marinum* wild-type cells is able to transfer mannose from an *in situ*-produced decaprenylphosphomannose donor to a synthetic Ara_6_ acceptor, but a membrane preparation of *M. marinum* Δ*MMAR_2439* does not show this enzyme activity (Appelmelk *et al.*, 2008). In contrast to this previous mutant, a membrane preparation from *M. marinum* Δ*MMAR_2380* was able to produce a mannosylated Ara_6_ acceptor similar to that of the wild-type strain (Fig. 2). This means that the arabinan mannose-capping enzymic activity is still present in the membrane of *M. marinum* Δ*MMAR_2380*; in other words, the gene product of *MMAR_2380* is not involved in the direct functioning of the LAM-capping mannosyltransferase.

### Structural differences between the glycan parts of wild-type and mutant LAM

**Fig. 3.**
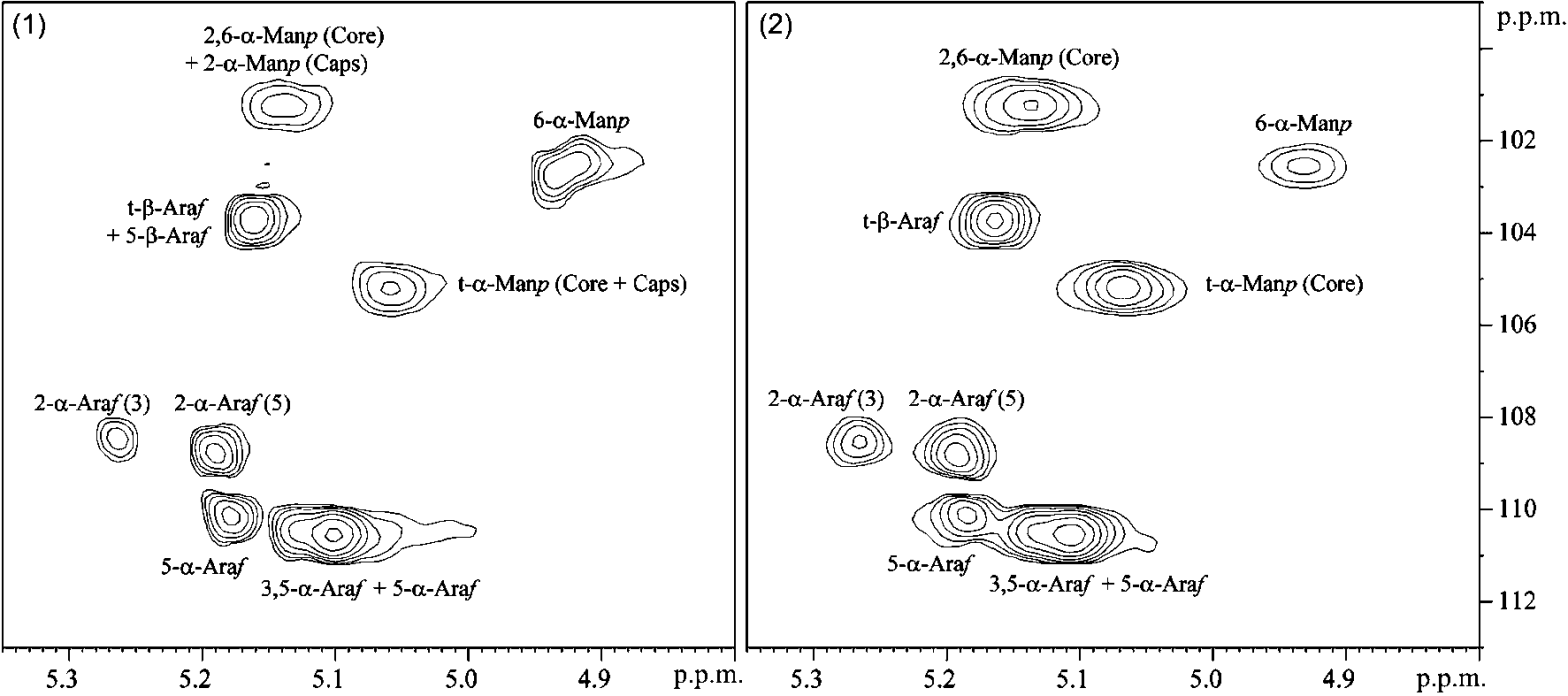
NMR analysis of the glyco part of LAM from *M. marinum* wild-type (pattern 1) and Δ*MMAR_2380* (pattern 2). 2D ^1^H-^13^C heteronuclear multiple quantum coherence spectroscopy (1H-^13^C HMQC) spectra of LAM in D_2_O at 313 K are shown with expanded regions (*δ* ^1^H, 4.80–5.35; *δ* ^13^C, 99–113). The different resonances are labelled with the abbreviated name of the corresponding glycosyl units. See Supplementary Fig. S1 for structure of ManLAM.

To investigate whether an alteration in the structure of the arabinan domain of mutant LAM might have caused the inability of the mutant strain to add the mannose cap, the glycan part of wild-type and mutant LAM was analysed by NMR. The NMR profile showed that the mutant LAM contained all mannosyl (Man*p*) and arabinosyl (Ara*f*) residues that are present in the wild-type LAM, even the single *α*(1→2)-linked mannoses added to the mannan core (Fig. 3). Most importantly, the *β*-arabinosyl residues which form the non-reducing termini of the branches of the arabinan domain and which are the acceptors for the mannose-capping enzyme MMAR_2439 (Supplementary Fig. S1), were still part of *M. marinum* Δ*MMAR_2380* LAM, and hence could, at least in theory, become substituted with a mannose cap.

Further analysis, however, did indicate an increase in the degree of branching of the mannan core in the mutant LAM, as revealed by the 2,6-*α*-Man*p* : (2,6-*α*-Man*p*+6-*α*-Man*p*) ratio, which was 78 % in the mutant and 28 % in the wild-type LAM. In a similar way, the arabinan domain of the mutant LAM seemed to harbour more lateral branches, with a 2-*α*-Ara*f* : 5-*α*-Ara*f* ratio of 0.35 in the mutant as compared with 0.29 in the wild-type LAM. Methylation analysis corroborated these conclusions, although the ratio values were somewhat different: the 2,6-*α*-Man*p* : (2,6-*α*-Man*p*+6-*α*-Man*p*) ratio was 50 % for mutant LAM and 36 % for the wild-type LAM, and the 2-*α*-Ara*f* : 5-*α*-Ara*f* ratio was 0.23 and 0.15 for the mutant and wild-type LAM, respectively.

Accordingly, in the biosynthesis of ManLAM, the function of the MMAR_2380 protein appears essential for the presence of the mannose cap, but its mutation also results in increased branching of both the mannan core and the arabinan domain of LAM.

### Acylation patterns of lipoglycans from wild-type and mutant strains

**Fig. 4.**
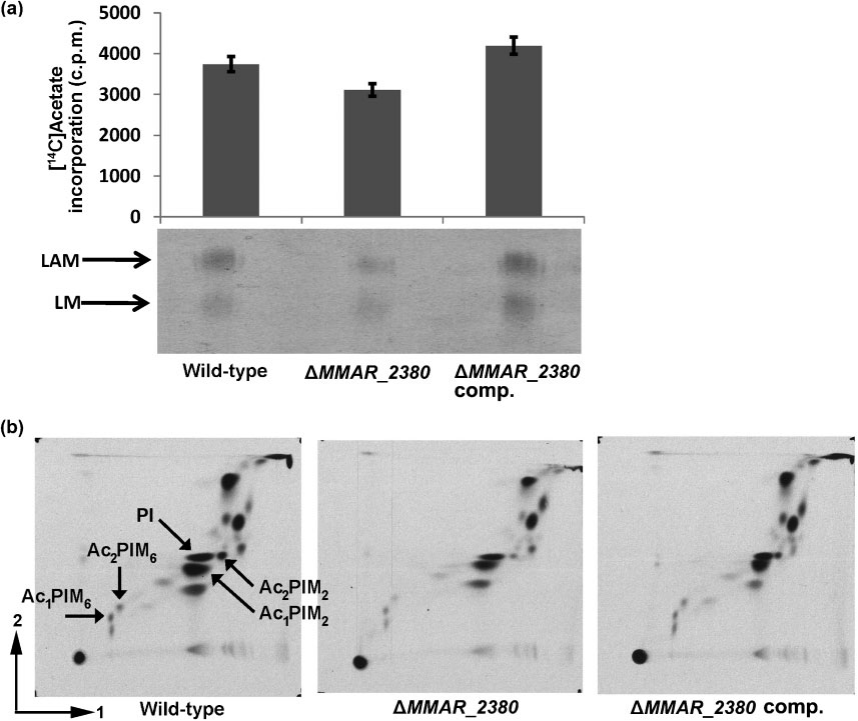
Reduced [1,2-^14^C]acetate incorporation in the polar lipids PIM_6_, LM and LAM from *M. marinum* Δ*MMAR_2380*. (a) Extracted crude lipoglycans from acetate-labelled delipidated cells (25 μg) from *M. marinum* wild-type, Δ*MMAR_2380* and complemented Δ*MMAR_2380* (Δ*MMAR_2380* comp.) were counted for the incorporation of [1,2-^14^C]acetate and analysed by SDS-PAGE/autoradiography. Shown is the average of two independent experiments. (b) [1,2-^14^C]Acetate-labelled *M. marinum* cultures were processed and polar lipids were applied (25 000 c.p.m.) to the corners of 6.6×6.6 cm pieces of aluminium-backed TLC plates and analysed using the 2D solvent system E as described in Methods. Plates were dried and autoradiograms were produced by overnight exposure of Kodak X-Omat AR film to the TLC plates to reveal [1,2-^14^C]acetate-labelled lipids. PI, non-mannosylated, diacylated phosphatidylinositol anchor; PIM_2_, phosphatidylinositol dimannoside; PIM_6_, phosphatidylinositol hexamannoside; Ac_1_PIM, tri-acylated PIM; Ac_2_PIM, tetra-acylated PIM. Unassigned spots in the figure are lipooligosaccharides, and in the upper-right corner are diphosphatidylglycerol, phosphatidylethanolamine and unknown phospholipids (Burguière *et al.*, 2005). Two independently prepared lipid extracts per strain were tested.

As MMAR_2380 is predicted to contain an acyltransferase domain, the acylation of mutant LAM was examined. To investigate this, *M. marinum* was cultured in the presence of [1,2-^14^C]acetate, after which lipoglycans were extracted and counted for the incorporation of [1,2-^14^C]acetate in the acyl chains (Fig. 4a). The data show that as compared with lipoglycans from wild-type *M. marinum*, lipoglycans from *M. marinum* Δ*MMAR_2380* have a reduced [1,2-^14^C]acetate incorporation. The complementation of *M. marinum* Δ*MMAR_2380* with wild-type *MMAR_2380* resulted in an increase of [1,2-^14^C]acetate incorporation. LAM and the related lipoglycan lipomannan (LM) were further examined by SDS-PAGE/autoradiography (Fig. 4a), which also confirmed the reduction of [1,2-^14^C] acetate incorporation in the mutant LAM and showed a similar effect for mutant LM.

As similar effects of mutation of *MMAR_2380* on [1,2-^14^C]acetate incorporation were seen for LM and LAM, we also analysed acylation of the structurally related phosphatidylinositol mannosides (PIMs). [1,2-^14^C]acetate-labelled lipid extracts were examined by 2D TLC using solvent system E for polar lipids (Besra, 1998; Dobson *et al.*, 1985), which indeed showed differences in the polar lipid profiles of the wild-type and mutant strains. The lipid extract from wild-type *M. marinum* showed [1,2-^14^C]acetate incorporation into phosphatidylinositol (PI), tri- and tetra-acylated phosphatidylinositol dimannosides (Ac_1_/Ac_2_PIM_2_) and phosphatidylinositol hexamannosides (Ac_1_/Ac_2_PIM_6_) (Fig. 4b). Interestingly, in the lipid profile of *M. marinum* Δ*MMAR_2380*, a subtle but reproducible reduction in [1,2-^14^C]acetate incorporation in both Ac_1_PIM_6_ and Ac_2_PIM_6_ could be observed (Fig. 4b). The incorporation of [1,2-^14^C]acetate in Ac_1_/Ac_2_PIM_2_, however, showed some variations between the experiments, and therefore was less clear.

Phosphatidylinositol dimannosides (PIM_2_) have been hypothesized to be precursors for phosphatidylinositol hexamannosides (PIM_6_) as well as LAM and LM, with phosphatidylinositol tetramannosides (PIM_4_) as a putative branching point (Besra *et al.*, 1997; Kovacevic *et al.*, 2006), and therefore alterations at or before this point of the biosynthesis pathway of LAM may carry forward into the final structure of LAM, as well as LM and PIM_6_. Because [1,2-^14^C]acetate incorporation analysis only provides information on potential quantitative differences between the wild-type and mutant lipoglycans, and to verify the results obtained from the TLC assays, we also subjected extracted PIMs to direct structural analysis by MALDI-TOF MS in an independent experiment. This technique can discriminate between the various forms of PIMs in terms of mannosylation and acylation (Gilleron *et al.*, 2006). The crude lipid extract from the mutant strain *M. marinum* Δ*MMAR_2380* contained all the different forms of PIMs present in the wild-type strain but with an apparent reduction of the most acylated forms (Supplementary Fig. S3), which might explain the decrease of [1,2-^14^C]acetate incorporation in the mutant.

Hence, while LAM, LM and all forms of PIMs are present in the mutant strain, small quantitative differences in [1,2-^14^C]acetate incorporation in PIMs, LM, and LAM between the wild-type and the mutant strain can be observed, which might arise from a reduction of the relative abundance of the most acylated forms.

### *M. marinum* Δ*MMAR_2380* has altered cell-surface properties

**Fig. 5.**
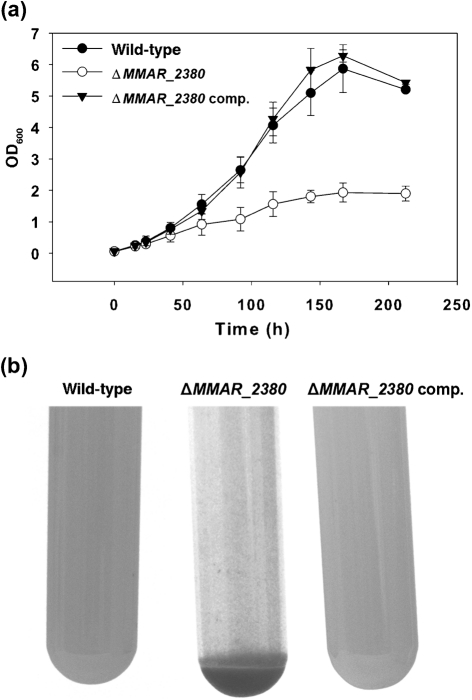
Physical appearance of liquid-grown *M. marinum* wild type, Δ*MMAR_2380* and complemented Δ*MMAR_2380* (Δ*MMAR_2380* comp.). (a) OD_600_ measurements during growth in 7H9+0.05 % Tween 80 with agitation. Shown are means of three independently grown cultures per strain; error bars sd. (b) *M. marinum* Δ*MMAR_2380* shows increased aggregation as compared with the wild-type and the complemented strains (see Supplementary Fig. S4 for colour pictures).

Along with the biosynthesis by *M. marinum* Δ*MMAR_2380* of LAM that is devoid of the mannose cap, another phenotypic trait was observed for this mutant strain. As compared with the wild-type strain, *M. marinum* Δ*MMAR_2380* showed severe aggregation. When grown as liquid cultures in 7H9 medium supplemented with 0.05 % (v/v) Tween 80, the mutant strain showed a reduced OD_600_ over time as compared with both its parent and the complemented strain (Fig. 5a). While the wild-type and complemented strain showed smooth yellow suspensions, the mutant strains showed aggregation and irregular pink clumps (Fig. 5b, and Supplementary Fig S4 for colour pictures). We cannot determine whether *MMAR_2380* is essential for optimal growth *in vitro* or, alternatively, whether the reduced OD_600_ in liquid cultures is solely due to aggregation. On 7H10 agar plates, the mutant strain showed the same colony morphology and growth rate as the wild-type strain (not shown). The effect of mutation of *MMAR_2380* on the cell-surface properties of *M. marinum* Δ*MMAR_2380* in liquid cultures was consistent with earlier reported results for a *Mycobacterium avium* mutant strain in which the homologous gene was mutated (Yamazaki *et al.*, 2006a, b). This *M. avium* mutant was impaired in its ability to produce large, wild-type amounts of biofilm (Yamazaki *et al.*, 2006b), which also negatively influenced its invasiveness of human bronchial epithelial cells (Yamazaki *et al.*, 2006a). On the other hand, in a designer arrays for defined mutant analysis (DeADMAn) screen of a transposon mutant library in *M. tuberculosis* CDC1551 for survival in mice, the mutant strain with a transposon in the homologous gene (Δ*MT1616*) was tested and did not appear to be attenuated in *in vivo* growth (Lamichhane *et al.*, 2005).

### The glycolipid profile and mannosylation pattern of proteins in *M. marinum* Δ*MMAR_2380* appear identical to wild-type patterns

The above-mentioned effect of the transposon insertion in *MMAR_2380*, the aggregation of the mutant strain, suggested a more pleiotropic effect on the bacterial surface, and hence could have affected, for example, the biosynthesis of glycolipids other than LAM. Other alterations in phenotypic traits, such as morphology, smooth/rough transition of colonies and biofilm formation, have often been linked to changes in glycolipid constituents (Belisle *et al.*, 1993; Belisle & Brennan, 1989; Daffé *et al.*, 1991; Howard *et al.*, 2006). Therefore, we performed an extensive lipid profile study in which polar and apolar extractable lipids were run in a range of four different TLC solvent systems, A to D, in addition to system E discussed above. Strikingly, no differences between the wild-type and the mutant strain could be observed (Supplementary Fig. S5).

Next, we checked for the mannosylation of proteins in *M. marinum* Δ*MMAR_2380* as compared with the wild-type strain, because of the specific role of MMAR_2380 in the mannose-capping of LAM. Whole-cell lysates were examined by an SDS-PAGE/immunoblot with the lectin concanavalin A (ConA), which recognizes *α*-linked mannose. Overall, the mannosylation pattern of proteins from *M. marinum* Δ*MMAR_2380* appeared similar to those of mannosylated proteins from the *M. marinum* wild-type strain, and the only difference observed in staining was at the position of (Man-)LAM on the blot (40–45 kDa) (Supplementary Fig. S6).

### Prediction of gene function

*MMAR_2380* encodes a 729 aa predicted transmembrane protein of unknown function (Supplementary Fig. S2). Therefore, a standard blastp search was performed against the nonredundant (nr) database at NCBI for the protein MMAR_2380 along with a search against the Conserved Domain Database (CDD) (Marchler-Bauer *et al.*, 2009). In contrast to the mannose-capping enzyme Rv1635c, MMAR_2380 (Rv1565c) is also present with high similarity in *Mycobacterium* species known to produce LAM without a mannose cap, e.g. in *M. smegmatis* mc^2^155 (MSMEG_3187; identities 75 %, positives 85 %). Its homologue in *M. tuberculosis* strain CDC1551 (MT1616) is annotated as putative lipopolysaccharide biosynthesis protein WbpC. Unfortunately, the exact function of the WbpC protein has not yet been determined. At present, WbpC is annotated as a possible *O*-acetyltransferase in the O-antigen biosynthesis cluster of *Pseudomonas aeruginosa* (King *et al.*, 2009). The N-terminal half of the protein contains a putative conserved domain of the acyltransferase-3 superfamily (Supplementary Fig. S2). As expected, many hits were found with predicted lipopolysaccharide biosynthesis acyltransferases from other non-mycobacterial species that contain the same conserved domain. MMAR_2380 was further predicted to be a protein with 10 transmembrane helices and a large C-terminal tail of approximately 300 amino acids in the periplasm (TMHMM 2.0) (Sonnhammer *et al.*, 1998).

Considering the effects of mutation of *MMAR_2380* described above, we postulate three hypotheses for how mutation of *MMAR_2380* may affect the biosynthesis of LAM and the related lipoglycans, LM and PIMs. The first possibility is that MMAR_2380 is a redundant acyltransferase involved in the acylation of LAM, LM and PIMs. The phosphatidylinositol synthase required for the MPI anchor is PgsA1 (encoded by *Rv2612c*; *MMAR_2090* in *M. marinum*) (Briken *et al.*, 2004; Jackson *et al.*, 2000), and acyltransferase Rv2611c has been shown to be involved in further acylation of PIMs (position C6 of the Man*p* linked to the C2 of the inositol) (Korduláková *et al.*, 2003). Importantly, the *Rv2611c*-mutant *M. smegmatis* strains still produce tri- and tetra-acylated PIMs (Korduláková *et al.*, 2003), and hence other acyltransferases are yet to be identified. From the TLC pattern (Fig. 4b), it is not possible to determine the position of acylation at which MMAR_2380 may be involved (position C3 of the inositol or position C6 of the Man*p* linked to the C2 of the inositol). However, the differences of [1,2-^14^C]acetate incorporation in PIMs, LAM and LM were small, and other effects of mutation of *MMAR_2380* were observed, suggesting a different function for MMAR_2380.

A second function for MMAR_2380 could be as a regulatory protein in the biosynthesis of LAM and related lipoglycans. The biosynthesis pathway of LAM has recently become better characterized. Not only glycosyltransferases directly involved in the addition of mannose and arabinose have been discovered, but also proteins with a role in the regulation of this pathway. One of these proteins is lipoprotein LpqW, which directs PIM_4_ into the biosynthesis route of LAM (Crellin *et al.*, 2008; Kovacevic *et al.*, 2006). Furthermore, the production of PIM_2_ and PIM_6_ has been shown to be compartmentalized in separate cell domains, a plasma membrane domain (PM*_f_*) and a plasma membrane–cell wall domain (PM–CW), respectively, each containing the enzymes required specifically for their biosynthesis (Morita *et al.*, 2005). Hence, as mutation of *MMAR_2380* resulted in a small quantitative decrease of [1,2-^14^C]acetate incorporation in mutant LAM as well as LM and PIM_6_ (Fig. 4), MMAR_2380 may also have a regulatory function in the metabolic pathway of these lipoglycans, e.g. in the recruitment of specific enzymes to the different membrane/cell wall fractions. In addition, as it is a regulatory protein, mutation of *MMAR_2380* could be the cause of the higher branching degrees of the mutant LAM, which could subsequently affect the LAM-capping activity in the mutant strain. Regarding the potential regulatory role of MMAR_2380, it has been reported that for certain enzymes additional processing such as acetylation is essential for proper transport and localization or for the activity of the enzyme (Banaei *et al.*, 2009; Fukuda *et al.*, 2002; Tschumi *et al.*, 2009), and MMAR_2380 may function in this way.

Finally, considering the altered growth of the mutant strain (Fig. 5), it cannot be excluded that mutation of *MMAR_2380* has a pleiotropic effect on the organization of the whole cell wall, and that it thus disturbs the compartmentalized biosynthesis of the higher-order PIM_6_, LAM and LM, and/or the transport of lower-order PIM_2_ from the PM*_f_* domain to the PM–CW fraction as precursor for the other lipoglycans, possibly resulting in the inability of the mutant strain to produce LAM with a mannose cap.

Summarizing, we have identified a novel gene involved, possibly indirectly, in the biosynthesis of the mannose cap on LAM, *MMAR_2380*, which is the *M. marinum* orthologue of *Rv1565c*. The mutant LAM was not only devoid of the mannose cap, but also displayed a higher degree of branching in both the mannan core and the arabinan domain as compared with wild-type LAM. In addition, mutation of *MMAR_2380* caused small quantitative differences in [1,2-^14^C]acetate incorporation in LAM, LM and PIMs, while all the different acylated forms of the PIMs seen in the wild-type strain were still present. Furthermore, another effect was seen: *M. marinum* Δ*MMAR_2380* showed increased aggregation in liquid cultures as compared with the wild-type strain, a phenotypic trait which was not observed for the previous mutant *M. marinum* Δ*MMAR_2439*, which produced ‘capless’ LAM. Hence, in all likelihood, *MMAR_2380* encodes an acyltransferase, although neither the length of the acyl chain transferred nor the acceptor can be predicted. Acylation activity by MMAR_2380 may be important for the biosynthesis of the mannose cap in a way which has yet to be determined.

## Abbreviations

- Ara_6_, hexaarabinofuranoside
- Ara*f*, arabinofuranosyl unit
- AraLAM, LAM with linked arabinosyl units
- BCA, bicinchoninic acid
- CE, capillary electrophoresis
- LAM, lipoarabinomannan
- LM, lipomannan
- ManLAM, mannose-capped LAM
- Man*p*, mannopyranosyl residue
- MPI, mannosylphosphatidylinositol
- PIMs, phosphatidylinositol mannosides
- PIM_2_, phosphatidylinositol dimannosides
- PIM_4_, phosphatidylinositol tetramannosides
- PIM_6_, phosphatidylinositol hexamannosides
- PM–CW, plasma membrane–cell wall domain
